# Persistent smoking after a cardiovascular event: A nationwide retrospective study in Korea

**DOI:** 10.1371/journal.pone.0186872

**Published:** 2017-10-19

**Authors:** Yoo Kyoung Lim, Dong Wook Shin, Hyeon Suk Kim, Jae Moon Yun, Jung-Hyun Shin, Hyejin Lee, Hye Yeon Koo, Min Jung Kim, Jeong Yeon Yoon, Mi Hee Cho

**Affiliations:** 1 Department of Family Medicine & Health Promotion Center, Seoul National University Hospital, Seoul, Republic of Korea; 2 Department of Family Medicine & Supportive Care Center, Samsung Medical Center, Seoul, Republic of Korea; 3 School of Nursing, Shinhan University, Uijeongbu, Republic of Korea; University of Tampere, FINLAND

## Abstract

Smoking is a major risk factor of cardiovascular disease (CVD) such as stroke and ischemic heart disease. Prior studies have observed people continued smoking even after being diagnosed with CVD. However, population-level data regarding smoking behavior changes among people who are diagnosed with CVD are still lacking. From the National Health Insurance sample cohort database, we identified 1,700 patients diagnosed as having CVD between 2003 and 2012, and underwent the national health screening examination in the year before and after the CVD event. We found that 486 (28.6%) were smokers before the CVD event. Among them, 240 (49.4%) continued to smoke despite the diagnosis. We observed that a higher smoking amount and longer smoking duration before the diagnosis were associated with persistent smoking. Our finding that approximately 50% of smokers continue smoking even after CVD events supports the need for an assessment of patients’ smoking statuses during follow-up after a CVD event and for health-care providers to offer the appropriate smoking cessation interventions to those who continue smoking.

## Introduction

Cardiovascular disease (CVD), including stroke and ischemic heart disease (IHD), is the leading cause of death across the globe [1]. In South Korea, the IHD mortality rate has been continuously increasing over the last 30 years, and since 2014, IHD became the second, after cancer, leading cause of death [2]. Although the incidence and mortality due to stroke have decreased, stroke was the most common cause of CVD until recently in Korea [3] and is still the third most common cause of mortality [4, 5]. In addition, CVD survivors are at a considerable risk of experiencing additional cardiovascular events [6]. For example, the cumulative risk of recurrent stroke is about 20–40% at 5 years after the first stroke [7, 8], and, among 1-year survivors of stroke, CVD is responsible for about 40% of all mortalities within the subsequent 4 years after the first stroke [9].

Smoking is a well-established risk factor for CVD. In 2012, The World Health Organization reported that 10% of all deaths due to CVD are attributed to smoking.[10] Moreover, persistent smoking increases the risk of recurrence and mortality in CVD survivors. Persistent smoking or smoking relapse doubles the risk of recurrence [11] and mortality [12, 13] in stroke survivors, and increases the risk of recurrence by about 50% [14] and doubles the risk of mortality [14] in IHD survivors. In contrast, several studies reported that smoking cessation decreases the incidence of subsequent events and the mortality rate in patients with CVD [14–16]. A systematic review by Critchley et al. reported that quitting smoking was associated with a 36% reduction in the risk of all-cause mortality among patients with IHD.[17] This is notable when compared to the risk reduction induced by other secondary prevention measures such as the intake of statins (29%) or aspirin (15%) [18, 19].

Previous studies have shown that despite the recommendation to stop or reduce smoking after being diagnosed with CVD, many patients continued smoking and their smoking habits varied considerably [20, 21]. A meta-analysis of 12 studies showed that the smoking cessation rates after myocardial infarction ranged from 29% to 74% [22]. The rate of persistent smoking following a diagnosis of IHD ranged from 7% to 63% in another meta-analysis study, which included 14 studies [21]. The rate of persistent smoking after a stroke was reported to be 50–80% [6, 23]. Moreover, findings for risk factors for continued cigarette use after CVD diagnosis are inconsistent. Some studies reported differences among the sexes with regard to smoking cessation, but others did not [21, 24, 25]. There has also been emerging evidence regarding of the influence of past depression on smoking cessation in general [26, 27], but such association was not found in Korean CVD population [28]. Most studies were limited by their cross-sectional nature, which is prone to recall bias [6, 24, 28, 29], and population-level data regarding smoking behavior changes among people who are diagnosed with CVD are still lacking.

The objective of this study was to assess the smoking status of patients before and after a CVD event and to determine the factors associated with persistent smoking. In this study, we used data from the National Health Insurance Service-National Sample Cohort (NHIS-NSC 2002–2013), a large retrospective population-based study [30]. The NHIS-NSC 2002–2013 was appropriate for our research because it included a large representative sample, data on the smoking status before and after the CVD event, and other demographic and medical characteristics.

## Methods

### Data sources

The Korean National Health Insurance (KNHI) is a mandatory universal public health insurance system that covers almost the entire Korean population except for Medicaid beneficiaries (approximately 3% of the population with the lowest income bracket). Medical providers, who are mostly in private practice, are reimbursed for their services basically on the basis of a fee-for-service scheme, and KNHI has all the information necessary for reimbursement of each medical service.

In addition, the KNHI also provides a biennial national health screening program to all KNHI beneficiaries older than 40 years and all employees regardless of age, and collects the information on the examination results.

The NHIS-NSC 2002–2013 is a cohort database available to the public for research purposes and contains deidentified medical claims data for 2% (n = 1,125,691) of the total KNHI population. The cohort participants were selected by random sampling in 2002; were stratified by age, sex, and income levels; and were followed up till the year 2013. The NHIS-NSC 2002–2013 comprises 3 databases: (i) eligibility database, (ii) medical treatment database, and (iii) health examination database. The eligibility database contains data on age, sex, income, residential area, disability, and mortality information. The medical treatment database includes data on the medical expense claims filed by medical service providers and the diseases treated. The health examination database contains the major findings uncovered during medical examinations and information related to lifestyle and behavior collected by administering questionnaires. The data from the KNHI database have been extensively used for epidemiological and health policy studies, and described in detail elsewhere [31, 32].

### Study population

We identified all patients who underwent a screening examination from the NHIS-NSC 2002–2013 register (n = 1,125,691). From these, we selected the records of 23,959 persons who were diagnosed with CVD between 2003 and 2012. We defined CVD as a diagnosis of CVD followed by inpatient hospitalization, as determined from the KNHI claim records. IHD included acute myocardial infarction (I21) and subsequent myocardial infarction (I22). Stroke included subarachnoid hemorrhage (I60), intracerebral hemorrhage (I61), other nontraumatic intracranial hemorrhage (I62), cerebral infarction (I63), and stroke that was not categorized as either hemorrhage or infarction (I64). The codes shown against each condition were obtained from the International Classification of Disease, 10^th^ Revision [33].

**Fig 1:**
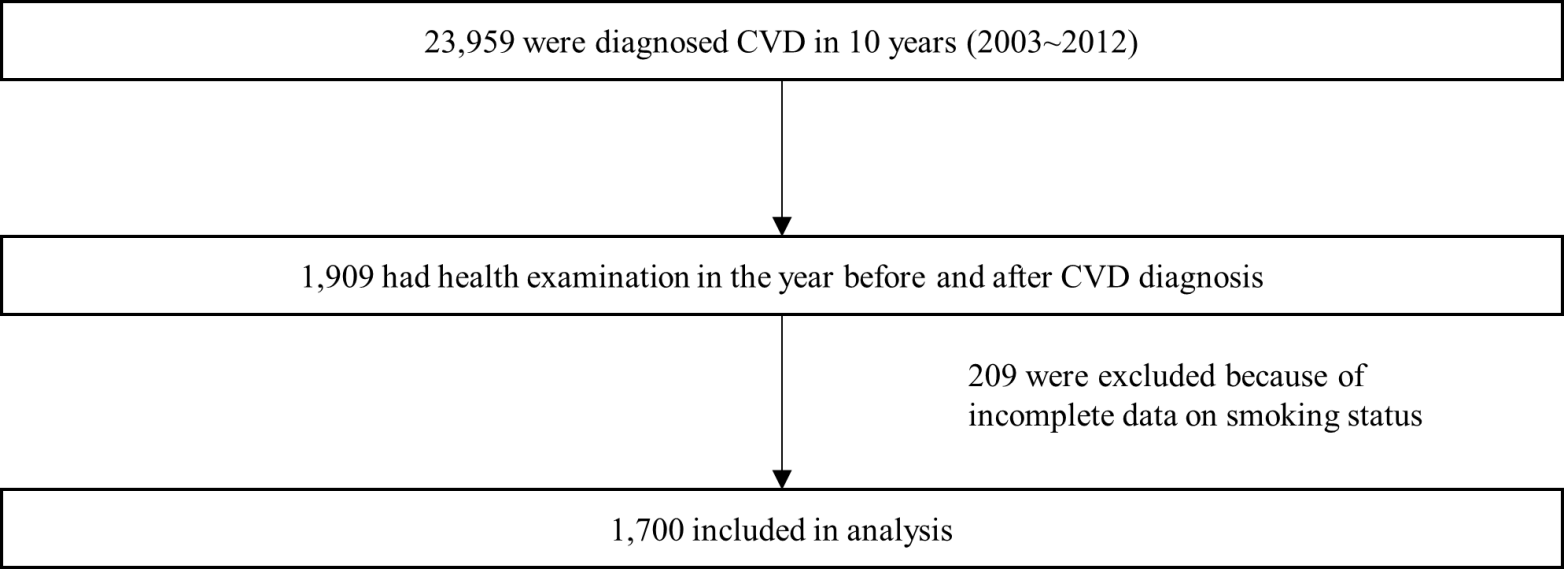
Flowchart for patient selection.

Health screening examinations are conducted biennially in Korea, and therefore we used 2-year intervals. Among those who were diagnosed with CVD, 1,909 underwent the national health screening examination both in the year before and after the CVD event (e.g., diagnosed with stroke in 2005 and underwent the health examination in both 2004 and 2006). After excluding participants who did not provide responses to questions on smoking status (n = 209), 1,700 participants were included in the final sample (Fig 1).

### Study outcomes

The primary outcome measure was the persistent smoking rate after a diagnosis of CVD, which was defined as current smoking in the year after the CVD event among those who reported current smoking in the year before the CVD event.

We obtained information on smoking status from the health examination database. People were asked to complete a self-administered questionnaire and provide categorical responses to questions on smoking status (i.e., non-smoker, ex-smoker, or current smoker). Ever smokers were also requested to provide information on the daily amount of smoking (number of cigarettes per day: <10, 10–19, 20–39, ≥40) and the duration of smoking (number of years smoking: <5, 5–9, 10–19, 20–29, ≥30 years). Body mass index (BMI) data were also obtained from the health examination database, and it was calculated as weight in kilograms divided by height in square meters. Because the insurance premium is proportional to the income level of the insured individual regardless of smoking status, we regarded the monthly insurance premium as the economic status.

### Statistical analysis

Differences in proportions were examined with the chi-square test. McNemar’s test was used to determine pre-post changes in smoking status for each CVD group. In addition, we used a multivariable logistic regression analysis to determine the factors associated with persistent smoking after a diagnosis of CVD, including age, sex, income, residential area, BMI, daily cigarette consumption, duration of smoking, and previous diagnosis with depression as potential predictors. As the insurance premium is determined to be proportional to the income level regardless of health status in Korea, we regarded the monthly insurance premium as proxy for the income level. Depression was defined by claims for treatment for depression (F32, F33) before the CVD diagnosis. All data analyses were performed by using STATA version 14.0. This study was approved by Seoul National University Hospital Institutional Review Board (No. E-1601-046-733), and informed consent requirement was waived as we used publicly open, anonymized data.

## Results

The characteristics of the study participants (n = 1,700) are described in Table 1. Among the participants, 486 (28.6%) were current smokers before the CVD event. Of the current smokers, 342 (70.4%) were diagnosed with stroke and 134 (27.6%) were diagnosed with IHD. A total of 10 participants had both stroke and IHD.

**Table 1:** Characteristics of study participants by smoking status before the diagnosis of cardiovascular disease.

	Smoker N = 486 (28.6%)^a^	Past smoker N = 194 (11.4%)	Nonsmoker N = 1020 (60%)	Total N = 1700 (100%)	p-value
Type of CVD					0.001
IHD	134 (27.6%)	53 (27.3%)	194 (19.0%)	381	
Stroke	342 (70.4%)	135 (69.6%)	805 (78.9%)	1282	
Both	10 (2.1%)	6 (3.1%)	21 (2.1%)	37	
Age(group)					0.000
20–39yr	96 (19.8%)	27 (13.9%)	74 (7.3%)	197	
40–49yr	148 (30.5%)	46 (23.7%)	176 (17.3%)	370	
50–59yr	136 (28.0%)	59 (30.4%)	311 (30.5%)	506	
60–69yr	93 (19.1%)	50 (25.8%)	345 (33.8%)	488	
70yr+	13 (2.7%)	12 (6.2%)	114 (11.2%)	139	
Sex					0.000
Male	463 (95.3%)	187 (96.4%)	432 (42.4%)	1082	
Female	23 (4.7%)	7 (3.6%)	588 (57.6%)	618	
Residential area					0.015
Metropolitan	202 (41.6%)	87 (44.8%)	365 (35.8%)	654	
City	206 (42.4%)	77 (39.7%)	435 (42.6%)	718	
Rural	78 (16.0%)	30 (15.5%)	220 (21.6%)	328	
Income					0.008
Income rank 1–2 (lowest)	83 (17.1%)	15 (7.7%)	167 (16.4%)	265	
Income rank 3–8 (middle)	173 (35.6%)	66 (34.0%)	323 (31.7%)	562	
Income rank 9–10 (highest)	230 (47.3%)	113 (58.2%)	530 (52.0%)	873	
BMI (Mean, SD)					0.096
	24.26 (2.94)	24.59 (2.72)	24.60 (3.04)		
Amount of smoking (cigarettes/day)					0.000
1–9	85 (17.5%)	N/A	N/A	N/A	
10–19	217 (44.6%)	N/A	N/A	N/A	
20–39	175 (36.0%)	N/A	N/A	N/A	
40+	9 (1.9%)	N/A	N/A	N/A	
Duration of smoking (years)					0.000
Yr < 5	10 (2.1%)	N/A	N/A	N/A	
5 ≤ Yr < 10	19 (3.9%)	N/A	N/A	N/A	
10 ≤ Yr< 20	75 (15.4%)	N/A	N/A	N/A	
20 ≤ Yr <30	130 (26.8%)	N/A	N/A	N/A	
Yr ≥ 30	252 (51.8%)	N/A	N/A	N/A	
Diagnosis of depression					0.000
No	452 (93.0%)	178 (91.8%)	883 (86.6%)	1513	
Yes	34 (7.0%)	16 (8.2%)	137 (13.4%)	187	

^a^ % is presented as row percentage

**Fig 2:**
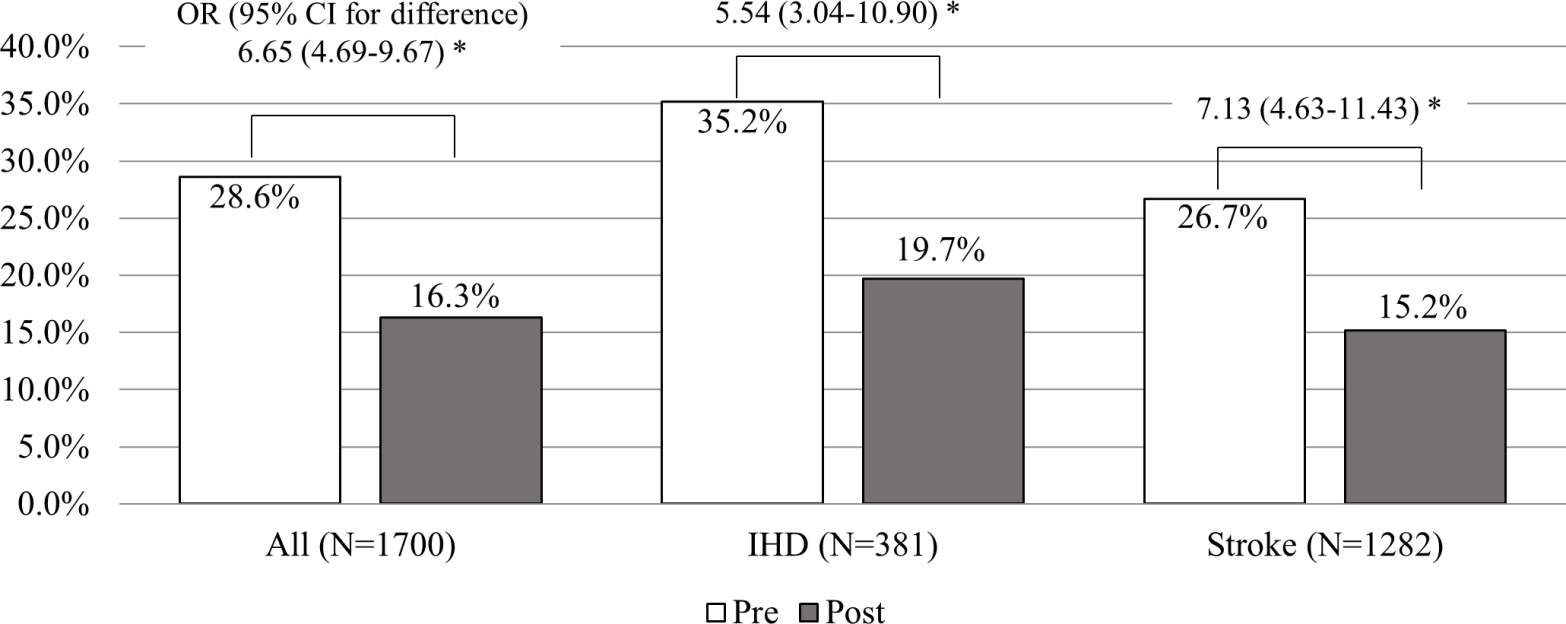
Comparison of current smoking rate between pre-diagnosis and post-diagnosis. * All differences were statistically significant by McNemar’s test (p < 0.001).

Fig 2 shows the proportion of smokers after the diagnosis of CVD in each CVD group subtype. The smoking rate decreased significantly after the CVD event: from 28.6% before diagnosis to 16.3% after the diagnosis (p < 0.001 by McNemar’s test). There was no significant difference in persistent smoking rates between smokers who had IHD (19.7%) and those who had stroke (15.2%) (χ^2^ = 2.09, p = 0.15).

The changes in smoking behavior observed in participants with each CVD subtype are described in Table 2. Among the 486 participants who were smokers before the diagnosis of CVD, 240 (49.4%) continued to smoke despite the diagnosis. Smoking resumption was reported by 6.7% (13/194) of individuals who had quit smoking before the CVD event. Twenty-four (2.4%) participants who were never smokers before the CVD diagnosis started smoking after the diagnosis. We also examine the amount smoked before and after the CVD event, and the number of cigarettes smoked per day has decreased after the event (S1 Table).

**Table 2:** Change of smoking status by subtypes of cardiovascular disease.

	Post-diagnosis smoking status		
Pre-diagnosis smoking status	Current	Past	None
All (N = 1700)			
Current (N = 486)	240 (49.4%)*	246 (50.6%)^§^	NA
Past (N = 194)	13 (6.7%)**	181 (93.3%)	NA
None (N = 1020)	24 (2.4%)***	NA	996 (97.7%)
IHD (N = 381)			
Current (N = 134)	62 (46.3%)*	72 (53.3%)^§^	NA
Past (N = 53)	4 (7.6%)**	49 (92.5%)	NA
None (N = 194)	9 (4.6%)***	NA	185(95.4%)
Stroke (N = 1282)			
Current (N = 342)	171 (50.0%)*	171 (50.0%)^§^	NA
Past (N = 135)	9 (6.7%)**	126 (93.3%)	NA
None (N = 805)	15 (1.86%)***	NA	790(98.14%)

*Persistent smoker

**Relapsed smoker

***Newly initiated smoker

^§^Quitter

NA: not applicable

We found that a higher smoking amount and longer smoking duration before the diagnosis were significant predictors of persistent smoking (Table 3). This association was evident only in the stroke group when subgroup analyses by disease type were performed. Age, sex, income level, residential area, BMI, and previous diagnosis with depression were not significant predictors of persistent smoking.

**Table 3:** Factors associated with persistent smoking after experiencing of cardiovascular events.

		All (N = 486)			IHD (N = 134)			Stroke (N = 342)	
	N	OR (95% CI)	P-value	N	OR (95% CI)	P-value	N	OR (95% CI)	P-value
Age									
15–39 (Ref)	96	-	-	30	-	-	65	-	-
40–49	148	0.95 (0.53–1.70)	0.86	47	0.57 (0.18–1.81)	0.34	99	1.00 (0.49–2.04)	1.00
50–59	136	0.97 (0.49–1.89)	0.92	33	0.51 (0.13–2.06)	0.35	98	1.04 (0.46–2.35)	0.92
60–69	93	0.84 (0.38–1.87)	0.67	21	0.18 (0.03–1.03)	0.05	70	1.08 (0.42–2.79)	0.87
70+	13	2.15 (0.53–8.75)	0.28	3	-	-	10	1.77 (0.37–8.49)	0.47
Sex									
Male (Ref)	463	-	-	128	-	-	325	-	-
Female	23	1.75 (0.64–4.76)	0.28	6	5.45 (0.53–56.44)	0.16	17	1.00 (0.49–2.04)	1.00
Income									
mid 60% (Ref)	173	-	-	51	-	-	119	-	-
lower20%	83	1.31 (0.77–2.23)	0.32	17	1.93 (0.58–6.47)	0.28	64	1.32 (0.70–2.50)	0.39
upper20%	230	0.76 (0.48–1.21)	0.24	66	1.70 (0.68–4.22)	0.26	159	0.59 (0.33–1.05)	0.07
Residential area									
Metropolitan (Ref)	202	-	-	58	-	-	139	-	-
City	206	1.09 (0.73–1.64)	0.68	61	0.95 (0.41–2.19)	0.90	141	1.17 (0.71–1.93)	0.53
Rural	78	0.93 (0.53–1.64)	0.80	15	1.19 (0.33–4.33)	0.79	62	0.88 (0.46–1.69)	0.70
BMI	486	1.01 (0.95–1.08)	0.67	134	1.04 (0.91–1.18)	0.61	342	1.00 (0.92–1.08)	0.99
Pre-diagnosis smoking amount (cigarettes/d)									
0–9 (Ref)	85	-	-	26	-	-	58	-	-
10–19	217	2.05 (1.15–3.63)	0.015*	56	1.00 (0.31–3.24)	1.00	156	2.50 (1.23–5.10)	0.012*
20–39	175	2.46 (1.33–4.56)	0.004*	50	1.36 (0.39–4.75)	0.63	121	2.86 (1.34–6.13)	0.007*
40+	9	2.00 (0.47–8.55)	0.35	2	0.82 (0.03–21.43)	0.91	7	2.48 (0.46–13.30)	0.29
Pre-diagnosis smoking duration (years)									
0–4 (Ref)	18	-	-	0	-	-	18	-	-
5–9	19	17.14 (1.69–173.36)	0.02*	4	0.12 (0.01–1.72)	0.12	15	14.48 (1.32–158.30)	0.028*
10–19	74	0.72 (0.78–57.75)	0.08	22	0.33 (0.03–3.99)	0.39	51	8.29 (0.93–74.22)	0.06
20–29	124	7.22 (0.86–60.45)	0.07	45	1.25 (0.09–16.81)	0.87	78	7.91 (0.92–68.31)	0.06
30+	251	12.18 (1.45–102.27)	0.02*	63	-	-	180	9.66 (1.12–83.10)	0.039*
Diagnosis of depression No (Ref)	452	-	-	126	-	-	316	-	-
Yes	34	1.07 (0.51–2.23)	0.86	8	0.70 (0.13–3.78)	0.68	26	1.18 (0.50–2.81)	0.70

* Separate analysis for patients diagnosed with both IHD and stroke was not displayed due to insufficient numbers.

## Discussion

The results of the present study suggest that many patients with a recent diagnosis of CVD change their smoking behavior. About 50% of smokers quit smoking following a diagnosis of stroke or ischemic heart disease. This observation supports health behavior theories that people change their smoking behavior in response to health problems possibly caused by smoking, such as stroke and IHD; that is, experiencing an adverse health event may motivate people to adopt risk-reducing health behaviors [34]. However, our results also imply that 50% of participants continued smoking even after the CVD event. Considering previous studies which showed intensive smoking cessation interventions during hospitalization increase smoking cessation rates [35], this finding is indicative of a lost opportunity to promote smoking cessation. In addition, considering that those who remain smokers even after a CV event have a more negative attitude toward health aspects and are less compliant with their medications [36], this population should be a target for intensive health education.

The persistent smoking rates in the year following the CVD event observed in our study were similar to those reported by a recent meta-analysis study (47% ±16%)[21]; however, the persistent smoking rates varied considerably from 7% to 63%, indicating the heterogeneity of the study population and design (i.e., prospective cohort, cross-sectional, and randomized controlled trial) and the timing of the post-diagnosis assessment (3 months to 13 years, mostly <2 years). A prospective Canadian study also reported similar persistent smoking rates among patients with IHD and stroke, and found that the rates declined from 17% to 9% and from 14% to 8%, respectively [37].

We compared our results with a previous Korean study, which examined the Korean National Health and Nutrition Examination Survey and reported that 63.6% of the patients who were smokers before the CVD diagnosis continued to smoke at the time of the assessment [28]. This number is somewhat higher than the persistent smoking rate observed in our study, and the discrepancy may be due to several factors: (i) the previous study was a cross-sectional survey of the general population and the mean time after CVD diagnosis was >7 years, indicating that people are more likely to resume smoking if more time has elapsed; (ii) the participants in our study were perhaps more health conscious because they underwent a regular health examination as compared to persons who did not undergo the regular health examination.

In addition, there were cases of smoking relapse among participants who had stopped smoking before the CVD event. The feeling of hopelessness or depression experienced by patients after the CVD event could have been a trigger for the smoking relapse. The prevalence of depression among patients with cardiac disease is about two to three times higher than that found in the general population [38]. Ex-smokers who are depressed are more likely to relapse as compared to the general population of smokers [39, 40]. Surprisingly, new smokers were identified in both groups (IHD: 4.6%; stroke: 1.9%). To our knowledge, previous studies have not reported on new smokers because the studies focused on participants who were smokers before the diagnosis. Further research is required to study the underlying reasons for this unique finding.

We did not find any association between change in smoking behavior and age, which is consistent with the findings of previous studies [28, 29]. There has been controversy with regard to the differences in persistent smoking between the sexes. Some studies have reported that the male sex was associated with an increased risk of persistent smoking [6, 41, 42]. In this study, no significant differences were observed between the males and females, which is consistent with the result of a recent meta-analysis study [21]. However, our study was limited in investigating sex difference as female smoking rate is low in our study population. In Korea, female smoking rate by self-report is only 6%, although that by biochemical marker is a little bit higher [43]. There was no significant association between previous diagnosis with depression and persistent smoking, either. We cannot rule out this might be due to the small number of smokers with previous depression, which was only 34 (7%).

Our findings indicated that the risk of persistent smoking was higher for patients whose tobacco consumption was high before the CVD diagnosis. Those who smoked >10 cigarettes per day were twice more likely to continue smoking after the CVD event as compared to light smokers. Participants who had a longer smoking duration at the time of the CVD event were more likely to continue smoking when compared to those who had smoked for <5 years before the CVD diagnosis. Our findings are largely consistent with previous studies, which reported that smoking fewer cigarettes per day and a shorter duration of smoking were predictors of successful quitting in the general population [44–46]. This implies that nicotine dependence also plays a key role in smoking behavior. However, in our study, such a trend was observed only among stroke patients and not among IHD patients, suggesting the effect of differences in patient condition or health services provided between the two CVD subtypes. One possible explanation is that the subjective perception of health status differs among patients with different CVDs. Symptoms of IHD usually include occasional pain and possible fear of death [47], and IHD patients who have a higher nicotine dependence might have a stronger intention to stop smoking in order to prevent the occurrence of a life-threatening health problem. However, strokes are often accompanied with neurologic sequelae, such as paralysis, and therefore stroke patients who have a higher nicotine dependence may develop a stronger desire to smoke despite the stroke-induced disabilities.

The strength of this study is that the data were obtained from a large, nationwide cohort of Korean people over a period of 12 years. Therefore, the magnitude of selection bias was reduced when compared to other single center-based studies. Furthermore, the use of the health examination and claims data reduced the recall bias because the pre-diagnosis smoking data were actually collected before the diagnosis and the diagnosis of CVD was based on findings during the actual healthcare examinations instead of medical history obtained through self-reporting.

This study suffers from several limitations. One limitation of this study is the possible misclassification of the self-reported smoking status by the participants. However, the effect of this misclassification would be not considerable because the health behavior of the participants is usually confirmed by doctors via interviews during the health examination. Second, our secondary data do not have the psychological or socioenvironmental profiles that could also affect smoking behavior after diagnosis such as marital status or education level [48–50]. Further studies are required to clarify additional risk factors of persistent smoking, which were not measured in this current study. Finally, we could not identify whether the smokers were offered any referral to a smoking cessation program or counseling after the CVD event. Many studies support individually delivered smoking cessation counseling to assist smokers to quit [51]. However, during the study period, smoking cessation services were not reimbursed by the KNHI and therefore were rarely offered to patients who were admitted for CVD. In 2015, the KNHI began to reimburse smoking cessation services, and further study could reveal whether routine provision of smoking cessation services could reduce persistent smoking in this population.

A diagnosis of CVD may be an opportunity for the initiation of secondary preventions or improving one’s health. Indeed, our findings indicated that a CVD event led to smoking cessation in about 50% of the smokers; however, the remainder continued to smoke even after the CVD event. Further, the risk of persistent smoking was increased in long-term smokers and heavy smokers. Health care providers should periodically screen the smoking status of patients during the follow-up after a CVD event and provide smoking cessation interventions.

## Supporting information

S1 Table The amount smoked before and after the cardiovascular event among persistent smokers (N = 243). (PDF)
