# Supplementary material for: Persistent smoking after a cardiovascular event: A nationwide retrospective study in Korea
Source: PLoS One. 2017 Oct 19;12(10):e0186872. doi: 10.1371/journal.pone.0186872 (PMC5648241; doi:10.1371/journal.pone.0186872)
Supplement: S1 Table — (PDF) [file pone.0186872.s001.pdf]

sTable 1. The amount smoked before and after the cardiovascular event among persistent smokers (N=243)

Pre-diagnosis smoking amount (cigarettes/d)	Post-diagnosis smoking amount (cigarettes/d)				
	0-9	10-19	20-39	40+	Total
0-9	19	13	2	0	34
10-19	23	64	22	0	109
20-39	4	39	48	4	95
40+	0	1	3	1	5
Total	46	117	75	5	243

P<0.05 by Friedman test
